# Falling living standards during the COVID-19 crisis: Quantitative evidence from nine developing countries

**DOI:** 10.1126/sciadv.abe0997

**Published:** 2021-02-05

**Authors:** Dennis Egger, Edward Miguel, Shana S. Warren, Ashish Shenoy, Elliott Collins, Dean Karlan, Doug Parkerson, A. Mushfiq Mobarak, Günther Fink, Christopher Udry, Michael Walker, Johannes Haushofer, Magdalena Larreboure, Susan Athey, Paula Lopez-Pena, Salim Benhachmi, Macartan Humphreys, Layna Lowe, Niccoló F. Meriggi, Andrew Wabwire, C. Austin Davis, Utz Johann Pape, Tilman Graff, Maarten Voors, Carolyn Nekesa, Corey Vernot

**Affiliations:** 1 University of California, Berkeley, Berkeley, CA, USA.; 2 The World Bank, Washington, D.C., USA.; 3 Innovations for Poverty Action, Washington, D.C., USA.; 4 University of California, Davis, Davis, CA, USA.; 5 Northwestern University, Evanston, IL, USA.; 6 Yale University and Y-RISE, New Haven, CT, USA.; 7 Deakin University, Melbourne, Australia.; 8 University of Basel, Basel, Switzerland.; 9 Stockholm University, Department of Economics; Research Institute of Industrial Economics, Stockholm, Sweden.; 10 Max Planck Institute for Collective Goods, Bonn, Germany.; 11 Jain Family Institute, New York, USA.; 12 Princeton University, Princeton, NJ, USA.; 13 Busara Center for Behavioral Economics, Nairobi, Kenya.; 14 Stanford University, Stanford, CA, USA.; 15 WZB Berlin, Berlin, Germany.; 16 Columbia University, New York, NY, USA.; 17 International Growth Centre, London, UK.; 18 Vyxer Remit Kenya, Busia, Kenya.; 19 American University, Washington, D.C., USA.; 20 University of Goettingen, Göttingen, Germany.; 21 Harvard University, Cambridge, MA, USA.; 22 Wageningen University & Research, Wageningen, Netherlands.

## Abstract

Despite numerous journalistic accounts, systematic quantitative evidence on economic conditions during the ongoing COVID-19 pandemic remains scarce for most low- and middle-income countries, partly due to limitations of official economic statistics in environments with large informal sectors and subsistence agriculture. We assemble evidence from over 30,000 respondents in 16 original household surveys from nine countries in Africa (Burkina Faso, Ghana, Kenya, Rwanda, Sierra Leone), Asia (Bangladesh, Nepal, Philippines), and Latin America (Colombia). We document declines in employment and income in all settings beginning March 2020. The share of households experiencing an income drop ranges from 8 to 87% (median, 68%). Household coping strategies and government assistance were insufficient to sustain precrisis living standards, resulting in widespread food insecurity and dire economic conditions even 3 months into the crisis. We discuss promising policy responses and speculate about the risk of persistent adverse effects, especially among children and other vulnerable groups.

## Teaser

Income, employment, and food security fell sharply across low- and middle-income countries during the COVID crisis.

## INTRODUCTION

As a result of the COVID-19 pandemic, economic activity has contracted around the globe. Fear of the virus and strict social distancing policies have led individuals in virtually all countries to modify their consumption and working habits. Economically vulnerable citizens of low- and middle-income countries (LMICs), where the majority of the world’s population resides, potentially face stark threats to their livelihoods. We use survey data systematically collected from 16 samples of over 30,000 households (containing more than 100,000 people) in nine countries in Africa, Asia, and Latin America to provide a rich, quantitative description of the economic effects of COVID-19 among various subpopulations in these LMICs.

There is reason to believe that rich and poor countries are experiencing the crisis very differently, which makes systematic documentation of the effects of COVID-19 in distinct settings critical. In industrialized nations, economic losses are often mitigated by government protection programs, employer adjustments to hours or compensation, or household savings. Absent broad social safety nets, declines in economic activity in LMICs can have more adverse welfare consequences, especially for those working in the informal economy. On the other hand, epidemiological models predict that health impacts of the virus may be weaker in LMICs given their relatively youthful populations (*1*). Poorer countries are also generally less connected to the global economy through trade and travel and, thus, were exposed to the pandemic later with valuable time to prepare and learn from the experiences of China, Europe, and North America (although it is unclear whether these opportunities were seized in practice) (*2*).

Goldberg and Reed (*3*) cite these factors to argue, using macroeconomic and financial statistics, that the initial economic effects of the pandemic were unexpectedly mild in LMICs. In contrast, international organizations have used similar aggregate data to make dire projections about gross domestic product (GDP) losses (*4*), decreases in remittance flows (*5*), and increases in poverty and hunger (*6*, *7*). Yet aggregate data have recognized deficiencies relative to direct surveying for tracking the well-being of the poor (*8*, *9*). In this study, we therefore rely on original, large-sample, representative household surveys to assemble a systematic and in-depth look at how the pandemic affected people’s lives in LMICs in the months following the global outbreak.

Household surveys are necessary because aggregate data can overlook large segments of the population. Over a quarter of economic activity and half of all workers in Africa, Asia, and Latin America are in the informal sector (*10*, *11*), and therefore are not captured in most official statistics. Informality similarly undermines the informativeness of measures of private sector transactions such as payroll, credit, or smartphone transfers. Hence, the approaches national statistical agencies and researchers [e.g., (*12*)] have used to document the economic losses from COVID-19 in industrialized nations cannot easily be implemented in LMICs. Many LMICs rely on periodic household or labor force surveys to measure economic activity. However, the low frequency of these surveys makes them insufficient for real-time tracking during a crisis.

Our research team rapidly adapted existing data collection protocols to deploy phone surveys starting in early April 2020 to track economic outcomes during the COVID-19 crisis. We use random sampling to generate statistically representative information about 16 populations in nine countries in Africa, Asia, and Latin America. The surveys cover heterogeneous samples constructed in different ways. Seven samples rely on random phone digit dialing (RDD) and skew toward wealthier and more educated mobile phone owners, while the other nine are drawn from earlier studies representative of specific subsamples, including formal and informal sector workers, agricultural laborers, small business enterprises, refugees, migrants, and their families. While the magnitude of effects varies across settings, the data reveal a consistent picture of heightened economic distress that spans both geography and socioeconomic strata.

The combined dataset documents steep drops in employment and income in our samples that rival or exceed economic losses experienced in the United States and other rich nations [e.g., (*13*, *14*)]. A full 50 to 80% of sample populations in Bangladesh, Burkina Faso, Colombia, Ghana, Kenya, Rwanda, and Sierra Leone report income losses during the COVID-19 period. If these effects persist, then they risk pushing tens of millions of already vulnerable households into poverty. This economic shock caused by the global pandemic spans socioeconomic strata within each country, and similar proportions of households at different rungs of the socioeconomic ladder report employment and income decreases. While the data shed light on the proportion of population suffering an income decline during the period, it should be noted that we are only able to characterize the magnitude of the decline for specific households in some of the samples.

By April, many households were already unable to meet basic nutritional needs. For example, 48% of rural Kenyan households, 69% of landless agricultural households in Bangladesh, and 87% of rural households in Sierra Leone were forced to miss meals or reduce portion sizes to cope with the crisis. Comparing to preexisting baseline data verifies that these levels greatly exceed the food insecurity normally experienced at this time of year. If anything, COVID-19 fortunately hit during a “postharvest” period in South Asia, when many people have grain stocks to draw down (*15*). High levels of food insecurity may continue to worsen as the crisis persists through the agricultural cycle.

The data paint a consistent picture: The economic shock and attendant disruptions to livelihoods during the early stages of pandemic appear to be large across a range of populations in Africa, Asia, and Latin America. The scale of the disruption may even exceed the effects that economists have documented in other recent global crises, including the 1997 Asian Financial Crisis, the 2008 Great Recession, and the Ebola outbreak of 2014 (*16*, *17*).

As a result, for LMICs, the economic crisis precipitated by COVID-19 may become as much a public health and societal disaster as the pandemic itself. The link from severe economic crisis during childhood to subsequent deterioration in adult health, nutrition, education, and earnings capacity has been demonstrated in many contexts. Almond (*18*) documents notable declines in education and adult earnings among those in utero during the 1918 influenza pandemic, and Maccini and Yang (*19*) show that children born during periods of weather-related economic hardship in Indonesia experience worse health, educational achievement, and income as adults. A growing body of long-run experimental research specifically links childhood nutrition to standards of living during adulthood [see (*20*, *21*)]. These channels of long-run transmission indicate that without mitigation, the substantial and widespread economic distress caused by the current pandemic may induce fallout that persists for decades into the future.

## DATA AND METHODS

### Sample construction

We present results from 16 samples in nine LMICs, as enumerated in Table 1. These countries—Bangladesh, Burkina Faso, Colombia, Ghana, Kenya, Nepal, Philippines, Rwanda, and Sierra Leone—have a combined population of nearly 500 million. We study several different subpopulations in Bangladesh and Kenya, and half of our study samples are drawn from two countries with a combined population of 200 million. In each sample, we conducted at least one telephone survey sometime during the period April–June 2020, after the outbreak of COVID-19 and the initial implementation of government lockdowns or other social distancing policies. Appendix A in the Supplementary Materials provides information on the timing of the spread of COVID-19 and government-imposed mobility restrictions relative to survey implementation dates across all countries in our sample.

**Table 1:** Description of household survey data samples used in the analysis.

**Country and COVID events**	**Projects**	**Households**	**Survey dates**
Bangladesh First case: March 8 Total cases (July 1): 149,258 Schools closed: March 17–August 6 Lockdown: March 26–May 30	BGD1. Rural sample: Rural households in villages participating in a project that aimed to increase access to the justice system	2229	May 2–12
	BGD2. Rohingya refugees from Myanmar: Refugee camp households in Cox’s Bazar district reported in (*35*)	367	April 11–17
	BGD3. Communities living near refugee camps: Host community households in Cox’s Bazar district	532	April 11–17
	BGD4. Participants in a lottery for agricultural work permits in Malaysia: Applicants for a temporary work program in 2013 in Chittagong and Dhaka Divisions	2936	April 16–20
	BGD5. Landless Rural Agricultural Laborers: Landless agricultural households in Northern Bangladesh first reported in (*36*, *37*)	294	May 31–June 2
Burkina Faso First case: March 9 Total cases (July 1): 962 Schools closed: March 26 Lockdown: March 21	BFA1. National sample (RECOVR): All adults with mobile phone numbers	1357	June 6–26
Colombia First case: March 6 Total cases (July 1): 95,043 Schools closed: March 24 Lockdown: March 24–July 1	COL1. National sample (RECOVR): All adults with mobile phone numbers	1507	May 8–15
Ghana First case: March 12 Total cases (July 1): 17,741 Schools closed: March 17–August 6 Lockdown: March 16–July 31	GHA1. National sample (RECOVR): All adults with mobile phone numbers	1633	May 6–22
Kenya First case: March 13 Total cases (July 1): 6366 Schools closed: March 20 Curfew: March 27	KEN1. Rural households in NGO cash transfer study: Households across 653 rural villages in NGO cash transfer study in Siaya County	8572	April 11–June 27
	KEN2. UNHCR refugees: All refugees and Shona stateless population with mobile phone numbers in Kenya	1332	May 14–July 3
	KEN3. Combined national sample: Phone numbers from the Kenya Integrated Household Budget Survey 2015/6 and all adults with mobile phone numbers	4052	May 14–July 3
Nepal First case: January 23 Total cases (July 1): 13,564 Schools closed: March 19 Lockdown: March 24+	NPL1. Agricultural households in western Terai: Rural households in the bottom half of the wealth in Kailali and Kanchanpur districts	1945	April 1–29
Philippines First case: January 30 Total cases (July 1): 37,514 Schools closed: March 17+ Lockdown: March 15+	PHL1. National sample (RECOVR): All adults with mobile phone numbers	1389	June 18–July 2
Rwanda First case: March 14 Total cases (July 1): 1205 Schools closed: March 16 Lockdown: March 21–April 1	RWA1. National sample (RECOVR): All adults with mobile phone numbers	1482	June 4–12
Sierra Leone First case: March 31 Total cases (July 1): 1462 Schools closed: March 31 Lockdown: April 5–7, May 3–5	SLE1. Candidate towns for rural electrification: Rural households taking part in an electrification program that installs solar mini-grids, described in (*38*)	2439	April 30–July 11
	SLE2. National sample (RECOVR): All adults with mobile phone numbers	1304	May 27–June 15

Nine of the samples (denoted BGD1–5, KEN1, KEN2, NPL1, and SLE1 in Table 1) in four countries were constructed from preexisting studies and were randomly drawn to be statistically representative of the population of interest for those earlier studies. An advantage of this is that we have additional pre–COVID-19 baseline data on living standards for these nine samples. When possible, survey questions were designed to be comparable to preexisting baseline data. In two of these samples (BGD5 and NPL1), we can compare the time path of outcomes to the typical seasonal pattern observed in prior years. The populations from which these samples are drawn vary, as described in Table 1 and in more detail in Appendix A in the Supplementary Materials.

Six other samples (BFA1, COL1, GHA1, PHL1, RWA1, and SLE2) are drawn via phone RDD, making them statistically representative of the set of active mobile phone numbers held by adults. Household surveys of mobile phone usage in various countries shows that the vast majority of adults in the sample countries and in LMICs more broadly now have access to mobile phones (*22*). For these samples, baseline data are constructed from respondent recall anchored to the introduction of substantial and memorable policy restrictions, typically the closure of schools. The final sample (KEN3) is a hybrid of 4052 households sampled from those in the 2015/16 Kenya Integrated Household Budget Survey who provided telephone numbers and an additional 767 adults contacted via RDD.

The primary reporting unit varies by sample. In samples constructed from existing study populations, household membership was already known, and the household is the unit of focus. A disadvantage is that this does not allow us to disaggregate all effects by gender, but some surveys collect outcomes specific to women and children (e.g., on domestic violence) that we will report below. In the RDD-based samples, data represent the individual adult associated with the phone number, with some limited questions about that individual’s household. Sampling weights for representativeness are described in Appendix A in the Supplementary Materials.

### Survey methods and timing

All post–COVID-19 data were collected via telephone interviews to minimize in-person contact and comply with government social distancing guidelines. Interviews were conducted by local enumerators in each country, with procedures to match languages, dialects, and accents between respondent and enumerator. Surveying by phone made rapid and large-scale data collection possible over large geographical units. In two samples (KEN1 and SLE1), we conduct high-frequency surveys spanning a long enough period to examine the evolution of post-COVID effects over time.

Unfortunately, interviewing by telephone places limits on data collection. Surveys were designed to be short, lasting only 15 to 30 min with relatively coarse measures of income and welfare, and render anthropometric measurements infeasible. Moreover, very poor households, who may not own phones or live in areas with low connectivity, may be underrepresented.

In the more agriculture-dependent nations in our sample, the baseline pre–COVID-19 period falls during a postharvest season in Bangladesh, Burkina Faso, Ghana, Nepal, and Sierra Leone, when food stocks in the rural population tend to be relatively high, and food prices are typically low. While this timing is fortunate for households’ ability to cope, postharvest is also a low point for agricultural labor demand in these countries, making subsequent declines in income and employment particularly notable. In Kenya, many farm households were already entering their “lean” season at the start of the crisis in March 2020. Natural seasonal variation in outcomes can complicate empirical inference on the true effects of COVID from these data, an issue we will address in greater depth below.

### Construction of outcome variables

Survey questions were coordinated across samples to the extent possible for data consistency. However, there were necessary adaptations for local context, and some questions were altered to conform to preexisting baseline data. To maximize consistency in reporting outcomes, we present each main result as the fraction of respondents reporting a change in an outcome post–COVID-19 relative to the preperiod. We provide a detailed description of how each variable was constructed for each sample in Appendix C in the Supplementary Materials and discuss the robustness of results to other reasonable ways of defining outcomes in Appendix B in the Supplementary Materials.

To examine heterogeneity of effects across socioeconomic strata, we further subdivide our analysis by socioeconomic status (SES) within sample. This classification is based on the within-sample median pre–COVID-19 consumption expenditure for six samples (BGD1–4, KEN1, and SLE1) and median pre–COVID-19 income for four samples (BGD5, KEN2–3, and NPL1). For the remaining six samples, high and low SES are distinguished by respondents’ scores on a “Poverty Probability Index” derived from the most recent national household survey, calibrated to either 100 or 200% of the national poverty line.

We calculate a “drop in income” measure at the household level in half of the samples (BGD1, BGD4, BGD5, KEN1–3, NPL1, and SLE1) and at the individual level in the remaining half (BGD2, BGD3, BFA1, COL1, GHA1, PHL1, RWA1, and SLE2). Four of the surveys (BGD2, BGD3, BGD5, and NPL1) compare income reported during pre–COVID-19 baseline surveys to income reported during the post–COVID-19 telephone interview to determine whether there has been a drop in income. The other surveys use retrospective reports of baseline income collected during the post–COVID-19 survey to compare with current income. We note that these retrospective reports carry the risk of respondent recall or reporting biases, which we would expect might lead to an overestimate of the extent of declines in income in some cases, a possibility we discuss further in the “Cross-survey comparability, representativeness, and limitations of the data” section.

We construct a “drop in employment” measure at the individual level in nine surveys (BGD2, BGD3, BFA1, COL1, GHA1, PHL1, RWA1, SLE1, and SLE2), with a drop defined as a respondent who reported working during the pre–COVID-19 reference period but not working at the time of the post–COVID-19 interview. Five surveys (BGD1, BGD4, and KEN1–3) measure the change in employment at the household level. In BGD1 and BGD4, a drop in employment is registered if any adult in the household was working during the baseline period but no adult was working during the post–COVID-19 period. In KEN1, a drop in employment is recorded if any adult in the household reports losing their job since February 2020 and is not currently working. The individual measures of employment declines are more strict than the household measures (with the exception of BGD1 and BGD4).

“Reduced access to markets” is measured at the household level based on respondent reports that they or any household member faced difficulties in purchasing food because of mobility restrictions, closed markets, or food shortages. Measures of food insecurity that we label “missed or reduced meals,” available for all samples, are based on respondents’ reports that they or someone in their households skipped meals or reduced portion size or quality. The reference period is the past 7 days unless otherwise noted in the appendix. In five surveys, households were classified as missing or reducing meals only if the respondent reported being unable to buy essential food items because of a lack of resources over the past 7 days (for BGD1–4) or 14 days (for NPL1). In the other surveys, there is no restriction on the reason for reducing or skipping meals.

Measures of “receipt of government or NGO support” are based on reports that the respondent (BFA1, COL1, GHA1, PHL1, RWA1, and SLE2) or the household (BGD1–5 and KEN1–3) have received food, cash, or other support from government programs or NGOs over the past month (BFA1, COL1, GHA1, PHL1, RWA1, and SLE1) or 2 weeks (KEN1–3). For BGD1–3, only households who reported being unable to buy essential food items over the past week because they lacked sufficient resources were asked whether they received assistance from government or NGOs. Table 2 reports this number as a share of the total sample, so is a lower bound estimate of the share of households that received such assistance in the case of BGD1–3.

**Table 2:** Change in living standards during the COVID-19 crisis in nine developing countries. This table shows statistics from 16 household survey samples in nine countries. Columns denote the share of households or individuals experiencing a (1) drop in income, (2) drop in employment, (3) reduced access to markets, (4) having health care access delayed, (5) having to reduce or miss the amount of meals, and (6) receiving NGO or government support. Column (7) shows the total number of households surveyed in each sample. Column (7) shows the maximum number of observations available for analysis in each study though specific measures are sometimes based on smaller samples. The division of respondents in each sample into “higher” and “lower” socioeconomic status is based on that respondent’s status within each sample, based on baseline consumption expenditure (BGD1–4, KEN1, and SLE1), baseline household income (BGD5, KEN2–3, and NPL1), and a Poverty Probability Index (others). Data from KEN1 are restricted to the first round of surveys. Blank cells denote that no data were available. These results are reproduced with standard errors in Appendix in the Supplementary Materials fig. S1.

	**Share of households experiencing:**						
	**Drop in** **income**	**Drop in** **employment**	**Reduced** **access to** **markets**	**Health** **care access** **delayed**	**Missed or** **reduced** **meals**	**Received NGO** **or government** **support**	**Number of** **households**
	**(1)**	**(2)**	**(3)**	**(4)**	**(5)**	**(6)**	**(7)**
**Bangladesh**							
BGD1. Rural sample	0.81	0.25	0.04	–	0.10	0.02	2229
Lower SES within sample	0.82	0.16	0.04	–	0.11	0.02	1102
Higher SES within sample	0.80	0.34	0.04	–	0.09	0.02	1127
BGD2. Rohingya refugees from Myanmar	0.44	0.31	0.31	–	0.27	0.26	367
Lower SES within sample	0.43	0.28	0.26	–	0.27	0.26	175
Higher SES within sample	0.44	0.35	0.37	–	0.27	0.26	192
BGD3. Communities living near refugee camps	0.73	0.16	0.25	–	0.23	0.02	532
Lower SES within sample	0.82	0.18	0.27	–	0.27	0.03	274
Higher SES within sample	0.64	0.14	0.23	–	0.18	0.01	258
BGD4. Participants in a lottery for agricultural work permits	0.71	0.29	0.10	–	0.09	0.02	2936
Lower SES within sample	0.72	0.29	0.10	–	0.10	0.03	1440
Higher SES within sample	0.70	0.28	0.10	–	0.09	0.02	1496
BGD5. Landless rural agricultural laborers	0.79	–	0.03	–	0.69	0.49	294
Lower SES within sample	0.70	–	0.04	–	0.74	0.52	145
Higher SES within sample	0.87	–	0.02	–	0.64	0.46	149
**Burkina Faso**							
BFA1. National sample (RECOVR)	0.63	0.29	0.49	0.11	0.28	0.25	1357
Lower SES within sample	0.69	0.32	0.56	0.09	0.35	0.19	631
Higher SES within sample	0.56	0.25	0.42	0.12	0.20	0.31	726
**Colombia**							
COL1. National sample (RECOVR)	0.87	0.49	0.68	0.43	0.59	0.28	1507
Lower SES within sample	0.95	0.51	0.71	0.41	0.75	0.43	217
Higher SES within sample	0.86	0.49	0.67	0.44	0.56	0.25	1290
**Ghana**							
GHA1. National sample (RECOVR)	0.84	0.33	0.30	0.11	0.52	0.22	1633
Lower SES within sample	0.86	0.30	0.35	0.11	0.50	0.19	654
Higher SES within sample	0.83	0.35	0.27	0.11	0.54	0.24	979
**Kenya**							
KEN1. Rural households in NGO cash transfer study	0.69	0.13	0.67	–	0.48	0.06	8572
Lower SES within sample	0.69	0.17	0.66	–	0.52	0.07	3171
Higher SES within sample	0.68	0.11	0.68	–	0.46	0.06	3148
KEN2. UNHCR refugees	0.08	0.30	–	0.15	0.56	0.11	1332
Lower SES within sample	0.07	1.00	–	0.15	0.56	0.11	1084
Higher SES within sample	0.12	0.06	–	0.13	0.55	0.08	248
KEN3. National sample	0.25	0.37	–	0.20	0.42	0.00	4052
Lower SES within sample	0.13	0.53	–	0.20	0.42	0.00	3139
Higher SES within sample	0.64	0.14	–	0.28	0.30	0.00	913
**Nepal**							
NPL1. Agricultural households in western Terai	0.39	0.19	–	–	0.11	–	1945
Lower SES within sample	0.34	0.18	–	–	0.13	–	800
Higher SES within sample	0.43	0.19	–	–	0.09	–	1145
**Philippines**							
PHL1. National sample (RECOVR)	0.52	0.42	0.77	–	0.35	0.40	1389
Lower SES within sample	0.58	0.50	0.81	–	0.41	0.43	364
Higher SES within sample	0.50	0.38	0.75	–	0.32	0.39	1025
**Rwanda**							
RWA1. National sample (RECOVR)	0.81	0.41	0.47	0.14	0.56	0.08	1482
Lower SES within sample	0.85	0.41	0.49	0.15	0.58	0.08	720
Higher SES within sample	0.76	0.43	0.44	0.13	0.53	0.09	762
**Sierra Leone**							
SLE1. Towns that are candidates for rural electrification	0.56	0.05	0.16	0.06	0.87	0.34	2439
Lower SES within sample	0.57	0.07	0.15	0.06	0.90	0.30	981
Higher SES within sample	0.56	0.05	0.16	0.06	0.85	0.37	1458
SLE2. National sample (RECOVR)	0.82	0.45	0.64	0.06	0.56	0.10	1304
Lower SES within sample	0.86	0.47	0.68	0.06	0.60	0.11	806
Higher SES within sample	0.71	0.39	0.54	0.04	0.46	0.07	498
Median share of respondents across samples	0.70	0.30	0.31	0.13	0.45	0.11	

Enterprise profit and revenue data in KEN1 are drawn from a parallel survey of a sample of enterprises drawn from a baseline pre–COVID-19 census of all enterprises in the villages where sample households reside. Current profits and revenues of enterprises are asked for the past 14 days; pre–COVID-19 profits and revenues are asked retrospectively for a “typical 2-week period in February 2020.” Enterprise profits in SLE1 are based on the household head’s self-employment profits over the past 7 days (post–COVID-19) or over a typical week in the month before the first lockdown.

Total consumption expenditure in KEN1 is based on the value of total household food consumption over the past 7 days and nonfood expenditure over the past 14 days. Consumption expenditure in SLE1 is based on household expenditure on five main staple food items over the past 7 days. To construct the pre–COVID-19 benchmark, the same expenditure question is asked retrospectively for a period about the same time, but a year prior.

To assess effects on consumer prices, we construct a price index for the KEN1 sample from respondent reports of the prices of 20 common consumer items. In the SLE1 sample, the price index is constructed from respondent reports on the prices of the five staples used to estimate consumption.

We also collected respondent reports of domestic violence in the KEN1 sample, in cases when female enumerators interviewed female respondents. After screening questions concerning privacy, safety, and willingness to discuss sensitive issues, respondents were asked three questions regarding threats of harm, physical abuse, and sexual activity being forced by any of their partners. Domestic violence against children is based on the respondent’s report that she or her husband/partner beat any of the children in the household. The reference period for all four questions is the previous 2 weeks.

### Cross-survey comparability, representativeness, and limitations of the data

The evidence is drawn from surveys of over 30,000 respondents in nine countries, with some coordination in questionnaire design. Yet the realities and constraints of survey work during the pandemic imply that none of the samples are nationally representative. In the seven RDD-based samples, the analysis is weighted to make the reported statistics representative of the active mobile phone numbers used by adults. While this is an increasingly broad portion of the adult population in these countries, it excludes people without access to mobile phones and may overrepresent people with multiple numbers. As a consequence, we see in a direct comparison of our survey respondents to nationally representative samples presented in Appendix table S1 in the Supplementary Materials that all of the RDD samples, with the exception of the Philippines, are much better educated than the populations of the countries from which they are drawn. In Burkina Faso, Philippines, Rwanda, and Sierra Leone the RDD samples are less poor, and in Burkina Faso, Ghana, and Sierra Leone they are more urban than the national population. Household sizes are larger in the RDD samples than in the national population in Ghana, Kenya, Philippines, Rwanda, and Sierra Leone. In the Philippines, RDD respondents are disproportionately female, while in Ghana they are more likely male.

Nine of the samples are based on earlier studies and are representative of specific subpopulations within a country. Pre–COVID-19 data from these studies provide an important baseline for our analysis. These samples are not intended to be representative of the entire country, as highlighted in Appendix table S1 in the Supplementary Materials. In Bangladesh, the populations sampled in our studies are very diverse. The Rohingya refugees (BGD2) and landless laborer (BGD5) populations are much poorer than the national average, while the communities living near the refugee camps (BGD3) are relatively better off than the national population. The other Bangladesh samples (BGD1 and BGD4) are both more rural and male than the population as a whole but have average incomes near the national average. The BGD1 rural sample has much lower education on average than the national population, while the BGD4 sample of applicants for agricultural work permits in Malaysia is better educated.

The Kenya rural household and refugee samples (KEN1 and KEN2), like the populations from which they are drawn, are both more rural and poorer than the Kenya national average. The rural KEN1 sample has much lower levels of education than the national average, and while the population of refugees also has lower levels of education, selection into the phone sample is such that the secondary school completion rate of the KEN2 refugee sample is similar to the national average. KEN3 households, in contrast, are more urban and better educated than the national average, but still poorer. Note that our Kenya samples are mostly located in regions that were unaffected by the 2019–2020 locust plague (*23*); when we exclude from our analysis the counties that were most affected by this plague, the results remain virtually the same (results available upon request).

The agricultural households surveyed in Nepal (NPL1) are all rural and much poorer than the national average. However, their secondary school completion rate is at the national average. The Sierra Leone Rural Electrification survey (SLE1) is entirely rural, consists of larger households than the national average, and has relatively few female respondents. The diversity of the samples we have gathered together makes comparisons across countries of the results more difficult. However, that same diversity provides some valuable insights into the impact of the COVID-19 pandemic across widely varying contexts.

In Appendix A in the Supplementary Materials, we characterize the timing of the first COVID-19 case and post-COVID survey dates in each country relative to the timing of the lean season. The post–COVID-19 reporting period in Burkina Faso, Kenya, Sierra Leone, and, to a lesser extent, Ghana occurs during the beginning of lean agricultural seasons. Some of the reported declines in employment, income, or food security that we observe may therefore reflect expected seasonal changes, rather than the effects of the epidemic. Current estimates of the extent of consumption seasonality in African contexts are that it is relatively modest. For example, (*24*) estimates a 2 to 3% decline in food consumption between the highest and lowest consumption months in Tanzania. There is, however, evidence that the quality and variety of food consumed vary more markedly across seasons than does the quantity of food (*25*). Our measure of food security focuses on quantities consumed (meals skipped or portion sizes reduced) rather than on quality and should therefore be less affected by seasonal changes.

As described more fully in Appendix B in the Supplementary Materials, some of the measures depend on respondent recall. For example, the “drop in income” variable collected in the seven RDD samples compares earnings over the past 7 days to pay in “a typical week” before government closed schools (or other marker of the onset of the COVID-19 crisis). These retrospective reports carry the risk of respondent recall or reporting biases based on their perception of the pandemic as a crisis, which could lead to an overestimate of the extent of declines in income.

In three of our samples (NPL1, BGD4, and BGD5), we can examine the extent of recall bias by comparing pre–COVID-19 outcomes as measured by survey responses elicited before versus after the onset of COVID-19. In Appendix B in the Supplementary Materials we report the three outcomes for which we have comparable data—pre–COVID-19 seasonal food security in the NPL1 sample, monthly household earnings in April in the BGD4 sample, and food security in January and February in BGD5—measured in surveys from both before and after the onset of COVID-19. In all three cases, the data about pre–COVID-19 outcomes as reported in our post–COVID-19 phone surveys closely track their counterparts as reported in pre–COVID-19 surveys. This consistency gives us some confidence that the pandemic itself did not have a large influence on recall-based reporting of prior conditions. However, this is still a difficult concern to address in general for all our data, and the possibility of these biases should be kept in mind.

## RESULTS

### Livelihoods during the COVID-19 crisis

Results in Table 2 document the widespread nature of economic hardships and the decline in living standards across the nine LMICs in the study. Across the 16 samples, between 8 and 87% of respondents report a drop in income during the crisis period, with a staggering median of 70% (column 1). The proportions reporting declines in employment are similarly high, ranging from 5 to 49% with a median share of 30% (column 2). The estimated magnitude of the economic shock remains stable whether comparing to preexisting baseline data or to respondent recall about their pre-COVID status as reported to us in a phone interview conducted after COVID hit. These measures capture the share of individuals or households that experienced a drop in well-being during the pandemic period rather than the net changes in income or employment. However, the proportion of respondents reporting declines in income (median 70%) exceeds those reporting rising income during the period by an order of magnitude (median across samples 7%). Appendix B in the Supplementary Materials discusses robustness of the estimates in detail.

The adverse economic shock experienced by individuals surveyed in these countries has been compounded by impediments to livelihood. In most countries, a large share of respondents report reduced access to markets, with the median share being 31% (range, 3 to 77%; column 3), likely related to the ubiquitous lockdowns and other mobility restriction policies adopted during March through June 2020. Where data are available, meaningful shares of respondents also report delays or other difficulties accessing health care (median, 13%; column 4).

Together, these drops in employment, income, and access to markets and services appear to contribute to higher levels of food insecurity. During the survey period, between 9 and 87% of respondents were forced to miss or reduce meals (median share, 45%; column 5), an issue we examine further in the next subsection. Even in Colombia (sample COL1), the country in our sample with the highest per capita GDP and thus potentially the greatest financial resources to cope with the crisis, the majority of respondents report drops in income (87%) and employment (49%), and an increase in food insecurity (59%).

Social support in response to the economic shock has been mixed in our populations of study. Across samples, the proportion of respondents who report benefiting from government or NGO crisis support runs the gamut from 0 to 49%, with a median of 11%. However, the high rate of missed meals and reduced portion sizes suggests that even when these efforts are present, they have been insufficient. For instance, Rohingya refugees in Bangladesh (BGD2) report the highest rates of assistance, given the preexisting international aid infrastructure serving those communities. Even in this sample, 27% of respondents report food insecurity. More detailed data in one sample (KEN1) indicate that households also engage in extensive dissaving, such as selling assets and spending stored cash, to stabilize consumption.

These adverse effects on employment, income, market access, and food security vary substantially both across countries and across different subsamples within countries. For example, in the subset of national surveys, the share of households experiencing a drop in income varies across countries from 25% in Kenya to 87% in Colombia. Within the Kenya samples, the share of households experiencing drops in income ranges from 8 to 69%. Thus, the median impacts shroud significant variation across settings. Especially within countries, it is likely that this heterogeneity results, at least in part, from differences across the subgroups surveyed.

At the same time, however, we find little evidence that this variation is systematic, e.g., by socioeconomic or refugee status. In most of cases, we cannot reject equality in the share of high and low SES households affected. However, the impact of an equivalent income drop may be greater among low SES households, as evidenced by the generally higher rates of food insecurity reported in these subsamples. There is similarly no clear pattern across refugee and nonrefugee populations. Levels of reported food insecurity are actually slightly lower among refugees than the host communities living near Rohingya camps in Bangladesh (BGD2 to 3). On the other hand, food insecurity is somewhat higher among refugees in Kenya compared with a national sample (KEN2–3). More detailed data collected in BGD2–3 surveys suggest that the presence of international humanitarian organizations in the Rohingya camp areas may have helped buffer the economic shock for refugees.

### Impact timing, magnitude, and seasonality

**Fig. 1:**
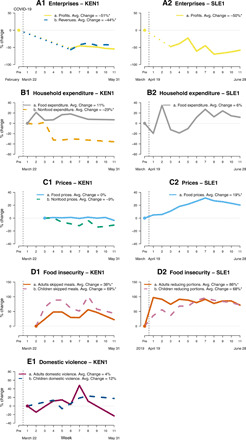
Evolution of key indicators over time. This figure shows the percentage difference from baseline for several indicators in rural Kenya and Sierra Leone during the COVID-19 global pandemic relative to the pre–COVID-19 or early COVID-19 levels. The Kenya sample is representative of all households and enterprises across 653 rural villages in three subcounties taking part in an unconditional cash transfer program. The Sierra Leone sample is representative of households in 195 rural towns across all 12 districts of Sierra Leone. Surveys in Kenya were conducted in two rounds. During the first round (weeks 1 through 8), 8594 households were interviewed. During the second round (week 11), 1394 households were surveyed, of which 1123 were interviewed for a second time. Surveys in Sierra Leone were conducted across 2439 households. The pre–COVID-19 levels are from questions that recall data from February (**A1**) and March (**A2** to **C2**) or from a previous survey conducted in November 2019 (**D2**). The post–COVID-19 levels are from questions that recall data from the prior 7 days (A to D2 and C to **D1**), prior 2 weeks (A1 and **E1**), and a combination (prior 7 days for food and prior 2 weeks for nonfood expenditures in B1). The weeks on the horizontal axis refer to the start of the recall period for each observation rather than the period during which the data were collected. The dotted lines in A1 and A2 show the linear trend from the pre-COVID baseline to the first observation for each respective time series. Baseline level for D1 is 1.3 days out of seven for adults and 0.72 for children. Baseline level for D2 is 35% of adults missing any meals in prior 7 days and 25% of children. Baseline level for E1 is 8% of adults experiencing violence in the prior 7 days and 20% of children. *P < 0.05.

We next describe the magnitude and timing of the effects on economic outcomes drawing on a subset of samples that feature more detailed panel or repeated cross-sectional data with richer measures of several key outcomes. Firm operations, a natural measure of overall local economic activity, appear to have been very adversely affected during the COVID-19 crisis where we have these data. In rural Kenya (KEN1), average firm profits and revenues dried up, falling by 51 and 44%, respectively (both with *P* < 0.05 relative to precrisis levels; Fig. 1A1). The analogous decline in Sierra Leone rural towns (SLE1) is a massive 50% (*P* < 0.05 relative to precrisis levels; A2). This evidence complements numbers on the share of the population experiencing any decline in employment or income in Table 2 by quantifying the depth of the economic decline.

In the rural Kenya sample, there is also a pronounced decline in per capita consumption expenditures during the crisis (B1), with declines in nonfood expenditures of 29% (*P* < 0.05 relative to the first observation period) persisting through all of April and May 2020. During the same period, food expenditures in Kenya and Sierra Leone actually rose slightly, by 11% (B1) and 6% (B2), respectively, although in Sierra Leone, this appears to have been driven by higher food prices facing these households (19%, *P* < 0.05 relative to the preperiod; C2) rather than greater quantities consumed. In contrast, Kenyan prices were largely stable or even fell slightly during the same period (C1). These data indicate that households appear to be cutting back nonfood consumption in an effort to maintain essential food intake.

Examining food insecurity in greater detail, we observe rising rates of missed meals and reduced portions during the crisis in both Kenya (D1) and Sierra Leone (D2), respectively. In Kenya, we record a 38% proportional increase in the rate of adults missing meals (0.5 meals per week) and 69% for children (0.5 meals per week). The proportional increase in the share of adults reducing portions in Sierra Leone is 86% (30 percentage points) and for children is 68% (17 percentage points, *P* < 0.05 for all of these effects). The sharp rise in food insecurity among children is particularly alarming given the potentially large negative long-run effects of undernutrition on later life outcomes (*26*, *27*).

The crisis period has been damaging for other dimensions of child development beyond nutrition. Schools in all sample countries have been closed during most or all of the study period. Nontrivial shares of respondents report reduced access to health facilities, including prenatal clinics and vaccinations (Table 2, column 4). The combination of a lengthy period of undernutrition, closed schools, and limited health care may be particularly damaging in the long run for children from poorer households who do not have alternative resources to make these critical human capital investments.

The rate of dissaving indicates there may be a range of other foregone household investments, from improved agricultural inputs to new small business opportunities. Lack of investment in both human and physical capital during a time of crisis can transmit the economic fallout of the pandemic far into the future.

The COVID-19 crisis could also have contributed to rising rates of domestic violence in the rural Kenya sample for which we have detailed survey reports (E1). Both violence against women and children—groups that are already marginalized in rural Kenyan society—rise by 4 and 13% (0.3 and 2.6 percentage points), respectively, during the crisis period, although these increases are not statistically significant. This increase in violence could generate additional negative and persistent effects on physical and mental health.

A central methodological concern in interpreting the patterns described in Table 2 and Fig. 1 is that factors other than the COVID-19 crisis could drive the evolution of outcomes over time. A leading possibility is that month-to-month seasonality, related for instance to the agricultural crop cycle, can also produce large changes over a span of a few months. It is challenging to fully address these concerns given distinct growing cycles for different crops in different countries, and sometimes even divergent harvest timings for different crops across regions within the same country. However, the consistency in outcomes across 16 different samples in nine countries on multiple continents, with a wide range of seasonal harvest and weather (and other) patterns, strongly suggests we are documenting the effects of a crisis that go beyond natural seasonal variation.

**Fig. 2:**
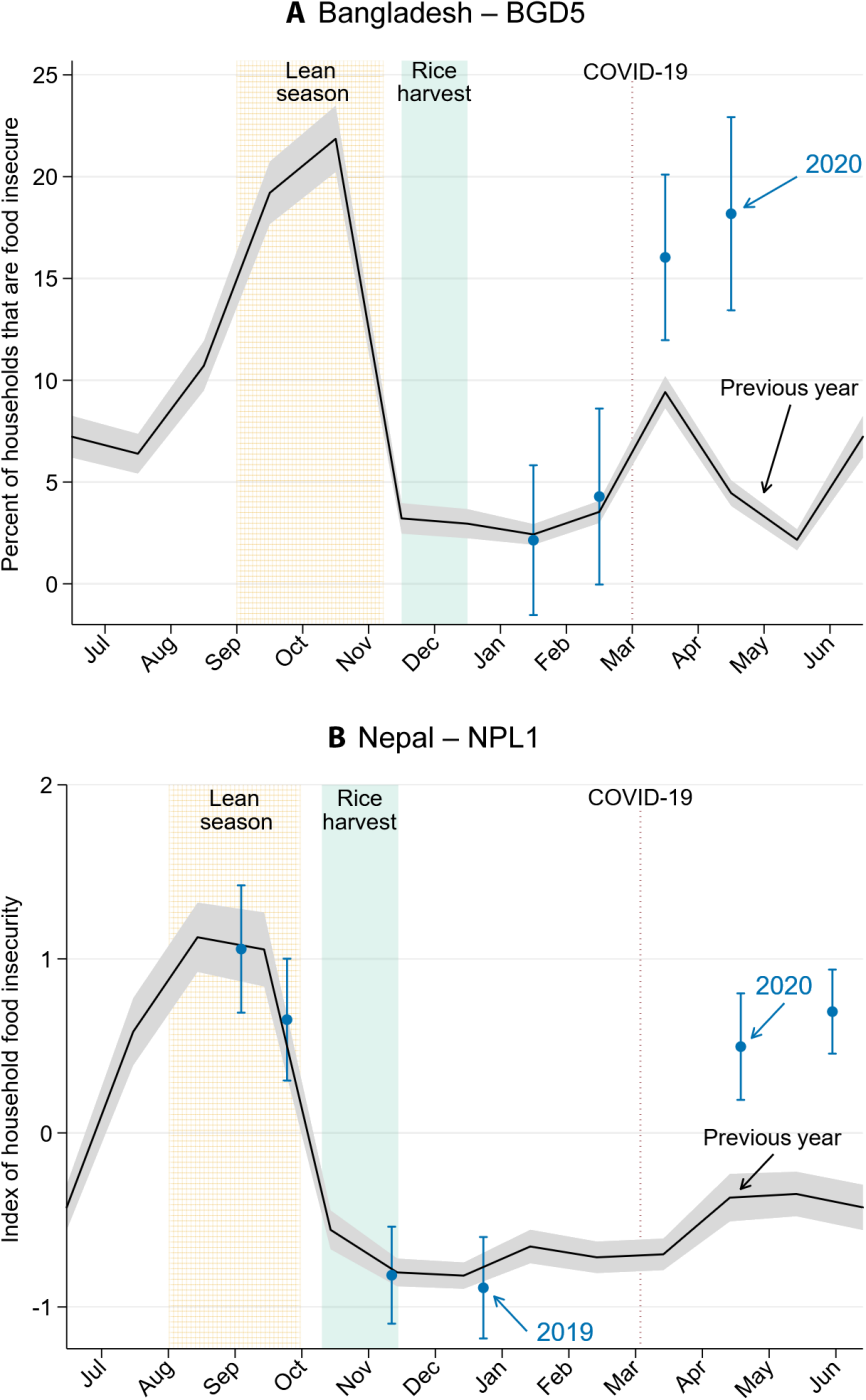
Food insecurity in Bangladesh and Nepal. Food insecurity in Bangladesh and Nepal with 95% confidence intervals. (**A**) Monthly rates of food insecurity among landless agricultural households in northern Bangladesh from sample BGD5. Food insecurity is defined as missing a meal or reducing portions for at least 15 days in a month. Note that this is a more stringent criterion than that reported in Table 2; in this figure, we restrict to cases of frequently missed meals. The 2020 rates come from an April phone survey, and “Previous year” reflects retrospective survey data spanning January 2018 through May 2019 collected in two survey rounds in February and June 2019. (**B**) Data from agricultural households in western Terai, Nepal, from sample NPL1. The index of food insecurity is constructed using two questions on how often households had to worry about not having enough food or had to reduce portion sizes. The data points in late 2019 and early 2020 come from six rounds of contemporaneous phone survey, and “Previous year” reflects respondents’ recollection about a prior “typical year” reported during the April–May 2020 phone survey round.

In two specific cases, we can directly contrast the excessive food insecurity experienced during the 2020 COVID-19 crisis to the natural seasonal patterns observed during those same months in previous years. In the BGD5 and NPL1 agrarian samples, there is monthly information on food insecurity during the 2016–2019 period that provides an ideal benchmark. Figure 2 clearly shows the pronounced seasonal variation in food insecurity in both Bangladesh (Fig. 2A) and Nepal (Fig. 2B) that spikes during preharvest lean or “hungry” seasons even during “regular” years. It is also apparent that levels of food insecurity are far higher during the 2020 crisis than they were during the same season in previous years: The rate of food insecurity in Bangladesh in April 2020 is roughly twice as high as in previous years, and this season-adjusted difference is statistically significant (*P* < 0.05). In these cases, the leading explanation for the effects we document in 2020 is the COVID-19 crisis rather than seasonal fluctuations. It is notable that in both countries, the COVID crisis occurred during the favorable postharvest period with its relatively low level of food insecurity during normal years. Baseline levels of deprivation typically rise sharply in the final 4 months of the calendar year.

## CONCLUSIONS AND IMPLICATIONS FOR POLICY

We document pronounced declines in employment, income, and food security since April 2020 across 16 survey samples in nine LMICs, with surveys covering over 30,000 households. This study provides some of the most systematic data to date on how the outbreak of COVID-19 has affected households across multiple LMICs in several major world regions. While the realities of rapidly deploying a survey over the phone during a pandemic make it difficult to reach a truly nationally representative sample, we study heterogeneous samples spanning three continents. We find that the economic shock in these countries—where most people depend on casual labor to earn enough to feed their families—leads to deprivations that seem likely to generate excess future morbidity, mortality, and other adverse longer-term consequences.

The findings highlight the importance of generating on-the-ground survey data to track well-being during the crisis to gather detail necessary to craft evidence-based policy responses. We demonstrate a path forward for gathering this information using large-scale phone surveys that rely on random sampling and standardized questions for comparability across settings. The methodology and harmonized measurement tools can readily be rolled out in new contexts to cover additional populations during this and future crises.

Following on decades of steadily increasing incomes across major world regions, the sharp rise in global poverty in 2020 that we document is unprecedented. The median proportion of respondents across our sample countries experiencing reduced income is a staggering 67%, and negative effects are experienced by households across the socioeconomic spectrum. The economic distress caused by the COVID-19 pandemic has had an immediate cost in terms of nutrition in LMICs. In addition to direct health consequences, hunger places long-run productivity and growth at risk as households compensate by reducing other investments in productive inputs such as fertilizer, selling productive assets, and lowering investment into long-run child development and education. Evidence abounds that these severe shocks to food security of children can threaten long-term health and well-being (*19*–*21*).

Humanitarian relief efforts that aim to address these problems face two added complications during the pandemic, relative to standard relief programs during regular years. The first is that further viral spread is fundamentally linked to the extent of economic deprivation, and successful disease containment requires the provision of immediate economic relief. Second, the worldwide financing of large-scale relief is constrained by the aggregate nature of the COVID crisis that simultaneously affected donor countries, as well as the large magnitude of the global economic recession.

The findings in our data highlight the first challenge. Households facing acute food shortages may be less willing to adhere to social distancing rules than others and could instead seek out income-generating opportunities even in crowded and epidemiologically risky markets. For social distancing to succeed, people must feel sufficiently secure from deprivation and hunger.

Relief programs should be carefully designed to avoid unintended adverse public health consequences—such as increased face-to-face market transactions in areas with high likelihood of viral spread. Cash or food transfers that allay this direct need could even double as tools to address disease spread by discouraging such market interactions. For example, transfers could be explicitly labeled with a “soft” form of conditionality, such as “this is money for food to reduce your need to work in crowded markets,” to further promote social distancing. Furthermore, new innovations to quickly and safely identify the poor using mobile phones or satellite data [e.g., (*28*)] and deliver funds remotely through mobile money transfers (*29*) hold promise in this context because of the minimal contact required to implement.

Our data also highlight the widespread nature of the global economic shock. Social protection programs in LMICs are underfunded even in good times. During an economic downturn, reduced tax revenue will make financing such programs even harder, and debt markets are not readily available for LMICs. Because the severity of the current crisis makes it important to expand safety net programs, international support—for instance, in the form of grants or concessionary loans—will be needed. Rich countries that are themselves under pressure from this same health and economic crisis may be tempted to focus on addressing problems at home. Yet, since disease transmission does not respect national borders, it is in the self-interest of wealthy countries to help reduce the spread of COVID-19 in LMICs, over and above any humanitarian motivations.

Policymakers in LMICs will also need to craft creative solutions to develop income-generating activities with longer gestation periods in case the risky COVID-19 disease environment or the associated economic slowdown persists for a prolonged period. For instance, “graduation programs” that combine assets and training can promote a source of livelihood that requires limited external contact and have been shown to reduce poverty in the past (*30*, *31*). Combining these programs with immediate cash support has even been shown to help build sustainable sources of income during periods of civil unrest [e.g., (*32*)].

On an optimistic note, the innovation and technological adoption that takes place during emergencies can spur long-run economic development. Dealing with the economic fallout from COVID-19 will require the technological infrastructure to reach poor populations in remote areas with minimal face-to-face contact. These workarounds have accelerated the expansion of new financial technology during past political and economic crises [e.g., (*33*, *34*)]. Solutions that arise in the current climate thus have the potential to both improve resilience immediately and durably advance the financial ecosystem.

Countries around the world face difficult policy choices along the path to economic recovery from COVID-19. While much public discussion focuses on “lives” and “livelihoods,” our data suggest this is a false dichotomy. We provide systematic evidence on how the outbreak has adversely affected households across multiple LMICs in several major world regions. A more appropriate framing of the situation in these countries could be in terms of “lives damaged or lost due to disease” and “lives damaged or lost due to economic deprivation.” We emphasize that our data do not speak to the economic consequences of imposing or relaxing specific lockdown policies. However, the evidence does have specific policy implications for how to cope with the economic hardships, to protect both lives now and in the future: fund and implement immediate humanitarian relief and long-term safety net programs to ameliorate the damage that we document.

## SUPPLEMENTARY MATERIALS

Supplementary material for this article is available at http://advances.sciencemag.org/cgi/content/full/7/6/eabe0997/DC1
